# Do United States' Teachers Know and Adhere to the National Guidelines on Asthma Management in the Classroom? A Systematic Review

**DOI:** 10.1155/2015/624828

**Published:** 2015-02-02

**Authors:** Yudilyn Jaramillo, Marina Reznik

**Affiliations:** ^1^Department of Pediatrics, Children's Hospital at Montefiore, Albert Einstein College of Medicine, 3415 Bainbridge Avenue, Bronx, NY 10467, USA; ^2^Yale University, Connecticut Mental Health Center, 34 Park Street, New Haven, CT 06519, USA

## Abstract

Proper asthma management in schools is important in achieving optimum asthma control in children with asthma. The National Heart, Lung, and Blood Institute (NHLBI) has developed guidelines on classroom asthma management. We conducted a systematic review to examine teacher knowledge of the NHLBI guidelines on asthma management in the classroom. We searched PubMed and EMBASE using search terms “asthma management,” “teacher(s),” “school teacher,” and “public school.” The inclusion criteria were articles published in English from 1994 to May 2014 that focus on schools in the United States (US). From 535 titles and abstracts, 9 studies met inclusion criteria. All studies reported that school teachers did not know the policies and procedures of asthma management. Teachers relied on school nurses to handle medical emergencies. Some studies identified that lack of full-time school nurses was a barrier to asthma management. Only one study showed directly that classroom teachers were not following the NHLBI guidelines on asthma management. Our literature review revealed that US teachers do not know the NHLBI guidelines on asthma management in the classroom. Future research should focus on interventions targeted toward training classroom teachers on asthma management as per NHLBI guidelines to ultimately improve asthma management in schools.

## 1. Introduction

Asthma is one of the most common childhood diseases that disproportionately burdens urban minority children [1–3]. Currently there is a significant difference in asthma prevalence between population subgroups. Overall, females have higher asthma prevalence than males, but in children aged 0–17, boys (11.3%) have higher asthma prevalence than girls (7.9%) [4]. Asthma is also a leading cause of school absenteeism in the United States (US) accounting for nearly 13 million missed school days per year [5]. Asthma prevalence is up to 20% in some urban schools [6].

Children spend up to half of their day at schools under the care and supervision of classroom teachers [7, 8]. Thus, classroom teachers may be the first to assist a student who is having an asthma attack in the school. Proper school asthma management can help a child with asthma achieve optimum asthma care [7, 8].

As per the National Heart, Lung, and Blood Institute (NHLBI) guidelines, if a school does not provide proper training to school staff on how to handle an asthma attack, students' asthma care may be jeopardized [9]. The NHLBI guidelines on asthma management for classroom teachers emphasize a strong family-physician-school partnership for optimal asthma management and on identifying children with uncontrolled asthma [9]. These guidelines have the following recommendations for the classroom teachers: classroom teachers need to (1) get updated on asthma policies and procedures through school nurses and school principals; (2) know their role when it comes to a student's asthma management (i.e., how to access a student's asthma action plan and have a copy of it in the classroom, maintained in a confidential manner; what steps to take in case of an asthma episode occurring in the classroom); (3) develop a clear procedure with a student and his or her parent(s) or guardian(s) for handling schoolwork missed if the student has episodes of illness or misses school; (4) encourage student's full participation in physical activity; (5) be aware of student's asthma triggers to let the nurse and parents know about them; (6) help reduce allergens and irritants that can provoke an asthma attack in the classroom; and (7) educate all students on lung health and asthma so students can be more understanding and accepting of classmates with asthma [9].

Individual studies have assessed teacher knowledge and perception about asthma management [10, 11]. However, teacher knowledge of the NHLBI guidelines on asthma management in the classroom has not been systematically reviewed. Thus, the aim of this systematic review of published peer-reviewed literature was to examine whether US know and follow the NHLBI guidelines on classroom asthma management.

## 2. Methods

### 2.1. Eligibility Criteria

This review included studies that reported on the role of classroom teachers managing students with asthma in the schools and the policies around it. The inclusion criteria were articles in English (published from 1994 to 2014) that addressed implementation of the NHLBI guidelines in US schools.

### 2.2. Information Sources

Our literature search used PubMed and EMBASE data sources. The search focused on titles and abstracts relevant to our research question for the period of January 1994 to May 2014. The last search was done on May 1, 2014. A hand search was done of the bibliographies from the retrieved articles.

### 2.3. Search Terms

**Figure 1:**
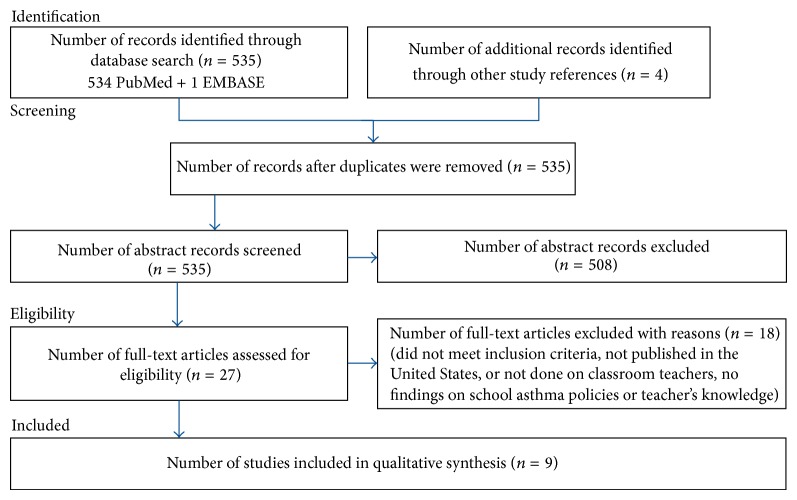
Flow chart of included studies.

The key terms used for this search were the following: “asthma management,” “teacher(s),” “school teacher,” and “public school.” One of the key term search combinations used was “asthma management and teacher and public school” which resulted in 7 articles using PubMed and 1 through EMBASE. Another key term combination used for the search was “school asthma management and teacher” which yielded 24 articles with PubMed and 0 using EMBASE. The last key term combination used was “asthma management and school teacher,” which resulted in 534 articles in PubMed and 1 in EMBASE (Figure 1). A title or abstract had to include asthma classroom teachers, nurses, or other school personnel to meet inclusion criteria. An article did not need to cite the NHLBI guidelines to be reviewed. After reviewing each research article found in PubMed and EMBASE, five articles met the inclusion criteria. In addition to the five pertinent articles, four more were found through the references from the selected articles. No filters were used during the search.

### 2.4. Article Selection

Articles found through the search were screened and selected in a 3-phase step process. In the first step we screened articles by study title and abstract, if available. Of the 535 total titles and abstracts found, 508 were excluded (Figure 1). The inclusion criteria were studies conducted in the US around asthma in public schools with school personnel as study participants. The second step was to read thoroughly each article that met criteria. This yielded 8 full articles to be included in our study analysis. We did a final third step of reviewing the reference lists from the articles that met criteria. Based on the articles' titles from the reference lists we found one additional article that met our inclusion criteria.

## 3. Results

### 3.1. Results of the Search

A total of 535 potentially eligible titles and abstracts were identified from the database searches and through the search of reference lists of included studies (Figure 1). Of these, 508 articles were excluded based on the title and abstract review, leaving 27 articles. After full text review, 9 articles met eligibility criteria. Reasons for exclusion were the following: non-US study, subjects not being teachers, and no description of teacher asthma knowledge and school asthma management or school health policies. The final 9 articles included in the review were comprised of cross-sectional/survey studies (*n* = 7), qualitative semistructured interview study (*n* = 1) and one intervention study using a quasi-experimental design.

### 3.2. Characteristics of the Studies

Table 1 describes characteristics of each of the nine included studies. The nine studies were all conducted in the US (3 studies were conducted in the state of Georgia [10, 12, 13], 2 in New York (NY) [11, 14], 1 in Nebraska and Wyoming [15], 1 in Iowa [16], 1 in Illinois [17], and 1 in New Mexico [18]). These studies were conducted mostly with elementary public school teachers but principals, school nurses, and other school personnel were also surveyed. Study outcomes are summarized in Table 2. Most of the studies (*n* = 8) revealed lack of teacher's asthma knowledge and training on asthma management in the classroom [10, 13–18]. The studies showed that teachers feel uncomfortable with managing asthma in their classroom because they are not trained to handle an asthma emergency [13, 16, 17]. Teachers viewed school nurses as a resource and relied on them to handle asthma or other medical emergencies [16, 17]. Two studies reported that lack of full-time school nurses to assist with student asthma management places the responsibility of managing asthma in schools on teachers [16, 17].

### 3.3. Study Types

#### 3.3.1. Cross-Sectional Studies

In the study by Neuharth-Pritchett and Getch, 291 of the 1000 surveys mailed to Georgia elementary school teachers were returned (29% return rate) [13]. The responding teachers reported receiving little training on chronic illness, asthma, or how to respond to medical emergency [13]. Neuharth-Pritchett and Getch also reported that when teachers were asked specifically about the number of teachers in their schools that had staff development training on managing asthma in the classroom, 84.9% stated that less than 25% of the teachers had training in asthma [13]. When questioned about their preparation to teach children with asthma, 77% indicated that they did not feel prepared [13].

Rodehorst conducted a study of 212 rural elementary school teachers from 19 schools in the eastern Wyoming and Western Nebraska [15]. Most or all of the teachers that participated were teachers from one grade level; only one physical education teacher participated [15]. Most of the teachers who lacked asthma knowledge (67%, *n* = 142) scored between 50 and 59% on the asthma knowledge questionnaire [15]. Only 4% (*n* = 10) scored between 80% and 89% [15].

A cross-sectional survey was done with 5 elementary schools in the Bronx, New York. Of 253 eligible participants, 156 (62%) responded. Survey participants included principals, assistant principals, teachers, counselors, and nurses [14]. In this study one interesting finding was that when assessing familiarity with existing Board of Education asthma policies, 64.1% of subjects reported that they were “unfamiliar” with the policy, 31.4% were “somewhat familiar,” and only 4.6% were “very familiar” with existing policies [14]. Also, this study found that 21% of subjects did not know who in the school was responsible for supervising the health needs of children with asthma [14].

Neuharth-Pritchett and Getch developed and evaluated the Teacher Capability and School Resource Scale for Asthma Management instrument designed to measure teacher capability, comfort, and resources regarding asthma management [12]. The instrument was developed from literature on school-based asthma management and asthma-related stressors [12]. This study showed that the scale constructed was a reliable tool to evaluate teachers' perception of how well they can handle the emotional or social aspects of a child's asthma at school and their perceptions of school and district resources available to assist in the management of children's asthma [12].

Getch and Neuharth-Pritchett explored teachers' knowledge of asthma and its management in a cross-sectional prevalence survey of a random sample of 2,000 public school teachers in Georgia. Of these, 593 teachers responded, including 291 elementary (*n* = 291) and middle-school (*n* = 302) teachers [10]. The study demonstrated that teachers who had asthma were more knowledgeable than those without the disease [10]. Even though a small percentage of teachers reported that they received asthma specific education through in-service training, the overall level of asthma knowledge remained low [10].

In another study, 320 pre-kindergarten through 5th grade classroom teachers who had at least one student with asthma in their classroom participated [11]. This cross-sectional study found that most teachers (54.5%; 84/154) took steps related to asthma trigger avoidance, and few took steps to coordinate medical care (5.2%; 8/154) or to monitor the students with asthma (7.1%; 11/154) [11]. Most teachers reported contacting or sending the child with asthma symptoms to the school nurse (65.4%; 87/133), with only 8.1% (7/87) of these teachers specifying the student was sent to the nurse for medication [11].

Lucas et al. conducted a cross-sectional study with 38 elementary school teachers (30 classroom teachers, 4 special education teachers, 2 physical education teachers, 1 music teacher, and 1 art teacher) [17]. The majority of teachers that were surveyed in the study (85.3%, *n* = 29) reported that they received no formal training on asthma during their coursework [17]. Only 32.4% (*n* = 11) reported having received training on asthma during teacher in-service trainings at their current place of employment [17]. Fifty percent (*n* = 17) of the teachers felt that they were unable to manage a child experiencing an acute asthma attack, with 29.4% (*n* = 10) feeling unsure of their capabilities [17].

#### 3.3.2. Qualitative Interview Study

The study by McCarthy et al. assessed NHLBI guideline adherence using semistructured interviews with 23 teachers (17 elementary teachers and 6 secondary teachers) and six principals (3 elementary principals and 3 secondary principals) from grades K-12 who were currently employed in urban or rural school districts in a Midwestern state [16]. All educators in the study reported experiences with children with chronic conditions. The most common disorders encountered were asthma, diabetes mellitus, seizure disorders, and cancer [16]. A frequently expressed concern by the educators was “fear that the educator might not know how to respond if one of the children had a medical emergency in the classroom or school” [16]. Some of the teachers also indicated that lack of full-time nurses was a barrier to asthma management [16].

#### 3.3.3. Intervention Study

A one-hour education intervention based on the NHLBI asthma guidelines was presented to 45 teachers at six New Mexico elementary public schools [18]. Pre- and posttests on general asthma knowledge, signs of respiratory distress on video footage, and comfort levels with asthma knowledge and medications were administered. General asthma knowledge median scores significantly increased postintervention (*P* < 0.0001). The ability to visually recognize asthma respiratory symptoms also significantly improved (*P* < 0.0001) [18].

#### 3.3.4. Adherence to the NHLBI Guidelines

Only one study conducted with 5 elementary schools in the Bronx, NY (156 eligible participants of whom 84% were teachers, 4% principals, and 1% school nurses) showed that classroom teachers were not following national guidelines on asthma management in the schools [14]. This study found that few teachers (2%) were “very familiar” with school asthma policies [14]. Only 10% of school personnel learned of a child's asthma through existing Board of Education protocols. In assessing familiarity with existing Board of Education asthma policies, only 1.6% of teachers reported being “very familiar” with existing school asthma policies, compared with 80% of principals (*P* < 0.05) [14].

#### 3.3.5. Lack of Nurse Staffing at Schools

Three studies out of 9 mentioned that there was a problem with nurses not being full-time on school grounds or not being always available [11, 16, 17]. Although some teachers felt less concerned because they had a nurse on school grounds, other teachers felt worried because although there was a nurse in school she may not be always available [16]. McCarthy et al. found that “most educators in the survey depended on school nurses to provide them with information about children with chronic conditions and to handle any problems related to these children” [16]. Lucas et al. showed that due to the shortage or absence of school nurse on school grounds the asthma management falls upon the teachers who interact with the child [17]. Another study described the lack of communication between the classroom teacher and the school nurse [11].

#### 3.3.6. Classroom Teacher Knowledge

In general, most of the studies (*n* = 8) showed that teachers do not have the knowledge of the school procedures for asthma management [10, 11, 13–17, 19]. In addition, teachers were not well trained to manage an asthma emergency if it was to happen [14, 17]. One study indicated that teachers were not adequately prepared to assist children with the management of asthma in the classroom [13]. Another study reported that even though a small percentage of teachers received an in-service asthma-specific education, the overall level of asthma knowledge remained low [10]. As per another study, nearly a third of the participants did not know how asthma inhalers were managed at their schools [14]. Similarly, in Roderhost study, the rural elementary school teachers' knowledge about asthma was low [15]. Lucas et al. found that half of the teachers in their study felt they were unable to manage a child experiencing an acute asthma attack, with some feeling unsure of their capabilities to deal with it [17].

#### 3.3.7. Classroom Teacher Confidence in Dealing with Classroom Emergencies

Teachers' confidence in dealing with health emergencies in the classroom was low. As per three studies, teachers did not feel comfortable dealing with a child with asthma [13, 16, 17]. Neuharth-Pritchett and Getch reported teacher fears over medical emergencies, medication administration, and liability issues that may have prohibited teachers from feeling comfortable with the classroom management of a child with asthma [13]. Teachers in this study reported that they received little exposure to coursework on chronic illness even though they indicated there was an average of four children with asthma per classroom [13]. McCarthy et al. found that educators were less confident in dealing with emergencies (asthma attacks, diabetes, or seizures) [16]. These educators were also concerned about what to do in a possible emergency situation and expressed a need for more training [16]. In Lucas et al. study some teachers expressed feeling unsure of their capabilities to deal with an asthma attack [17].

## 4. Discussion

Proper asthma management in schools is an important step to help a child with asthma achieve optimum disease control. Classroom teachers spend most of the school hours with the students than any other school personnel and with proper training can help with student asthma management. With nurses not always available on school grounds, it is important to have classroom teachers knowledgeable and well trained to manage an asthma emergency if it was to occur.

This systematic review revealed that many teachers were not well trained or did not know school policies on asthma management of students. While on average a classroom teacher may have up to four children with asthma in their classroom, teachers either received very little or no in-service training on classroom asthma management [10, 13, 15, 17, 20]. This may affect a child with asthma care at the school. Untrained teachers were fearful of any emergency medical conditions, including asthma, and may delay treatment [13]. These teachers indicated that they did not feel prepared to teach students with asthma. The researchers did not know whether teachers' lack of training was associated with the fear of assisting children with medical issues such as asthma [13]. Nurses could be the source of training for teachers on managing asthma in the classroom and may help alleviate teachers' fears of handling an asthma attack in the classroom. Some studies showed that another problem with the asthma management in schools was not having full-time nurses on school grounds [11, 16, 17]. This means that school staff, including classroom teachers or administrators, may need to handle asthma exacerbations and medication administration if an asthma emergency was to happen [21].

This study had several limitations. We did not include gray literature, such as unpublished work and dissertations, in our search due to limited resources. While most of the studies (7 out of 9) employed a cross-sectional study design and measured teachers' asthma knowledge, these studies used different instruments to assess knowledge and evaluated other outcome measures. These differences limited comparisons between the studies. Due to heterogeneity of the outcomes, we could not assess publication bias or perform a meta-analysis.

Little is known if US teachers adhere to the NHLBI guidelines on asthma management in classrooms. Our systematic review suggests that there was poor teacher adherence to these guidelines. School-based interventions and policy changes are needed to help increase awareness and use of the NHLBI guidelines in schools. Intervention programs to train classroom teachers on asthma management as per national guidelines may optimize students' asthma care at the schools. Such interventions should be developed, standardized, and evaluated in future research.

To improve classroom teachers' asthma knowledge and confidence in managing an asthma attack, an on-going required in-service training (e.g., at the beginning of each school year) may be needed. With proper training, classroom teachers could be at the forefront of managing an asthma emergency, especially in schools with limited availability of nurses. Teachers knowledgeable of identifying asthma symptoms and confidence in managing an acute asthma attack in a classroom would mean less missed school days and better asthma care.

## Figures and Tables

**Table 1:** Study characteristics.

Primary author (year)	Sample size	Population	US state study was done	Study design
Neuharth-Pritchett and Getch (2001) [13]	291	Public elementary school teachers	Georgia	Cross-sectional survey

Rodehorst (2003) [15]	212	Rural elementary school teachers	Western Nebraska and Eastern Wyoming	Cross-sectional survey

Snow et al. (2005) [14]	156	Principals, teachers, and school nurses	Bronx, New York	Cross-sectional survey

Neuharth-Pritchett and Getch (2006) [12]	589	K thought 8th grade teachers	State of Georgia	Cross-sectional survey

Getch and Neuharth-Pritchett (2009) [10]	593	Teachers from elementary and middle school.	Atlanta, Georgia	Cross-sectional prevalence survey

Bruzzese et al. (2010) [11]	320	Pre-kindergarten through 5th grade classroom teachers who had at least one student with asthma in their class	5 boroughs of New York City	Cross-sectional survey

Lucas et al. (2012) [17]	38	Elementary school teachers	In a rural school district of Illinois	Cross-sectional survey

McCarthy et al. (1996) [16]	23	Teachers (17 elementary teachers and 6 secondary teachers) and six principals	Midwestern state	A descriptive exploratory qualitative study

Sapien et al. (2004) [18]	45	Elementary public school teachers	New Mexico	Quasi-experimental pre-/posttest intervention study

**Table 2:** Study outcomes.

Primary author (Year)	Study outcome
Neuharth-Pritchett and Getch (2001) [13]	Teachers were not well prepared to help children with asthma in the classroom. Teachers reported that they did not receive enough workshops on chronic illness even though they identified that on average they have 4 children with asthma in the classroom. Teachers did not feel prepared to teach children with asthma. Fears over medical emergencies, medication administration, and liability issues may prohibit teachers from feeling comfortable with the classroom management of a child with asthma. National guidelines were not followed; teachers were not trained.

Rodehorst (2003) [15]	Teachers' knowledge from rural elementary schools about asthma was low. Very few teachers identified the actions to take. The intention to manage children with asthma in the classroom was very high. Teachers who had asthma did not have a higher knowledge than those who did not have asthma. Teachers may feel vulnerable in caring for a child with asthma; additional support from other coworkers appeared to be beneficial. Poor adherence to national guidelines; teachers were not knowledgeable.

Snow et al. (2005) [14]	Poor adherence to the national guidelines and lack of consistency on handling an asthma attack by teachers. National guidelines were not followed.

Neuharth-Pritchett and Getch (2006) [12]	The instrument researchers developed was found to be reliable in evaluating teacher capability, comfort, and resources regarding asthma management.

Getch and Neuharth-Pritchett (2009) [10]	Teacher knowledge on asthma was low regardless of teacher's educational background, whether teacher had asthma or not, and whether the teacher worked at elementary or middle school. Teachers received little training about asthma and its management. By providing asthma education to teachers they could become leaders in managing asthma in the schools. National guidelines were not followed.

Bruzzese et al. (2010) [11]	The majority of teachers correctly identified potential triggers, with the exception of cockroaches and laughing. Most teachers 54.5% (84/154) took steps related to trigger avoidance, and few took steps to coordinate medical care 5.2% (8/154) or to monitor the children. Most teachers reported contacting or sending a child with symptoms to the school nurse 65.4% (87/133), with only 8.1% (7/87) of these teachers specifying the student was sent to the nurse for medication. Only 18.0% (24/133) notified parents when students had symptoms in school. Almost half the teachers 46.7% (148/317) reported initiating communication with the school nurse regarding asthma; of these, 95.3% (141/148) described the nature of the communication. Although teachers reported discussing between one and three topics, the vast majority 81.6% (115/141) initiated discussion on only one topic. National guidelines were not followed.

Lucas et al. (2012) [17]	The majority (85.3%, *n* = 29) reported that they received no formal training on asthma during their coursework. Only 32.4% (*n* = 11) reported having received training on asthma during teacher in-service at their current place of employment. Fifty percent (*n* = 17) of the teachers felt that they were unable to manage a child experiencing an acute asthma attack, with 29.4% (*n* = 10) feeling unsure of their capabilities. Twenty-nine (85.3%) of the participants reported that it would be helpful to have educational sessions on asthma. National guidelines were not followed.

McCarthy et al. (1996) [16]	The concern most frequently expressed by the educators was fear that the educator might not know how to respond if one of the children had a medical emergency in the classroom or school. Teachers described possible emergencies as seizures, asthma attacks, or diabetic reactions. Some of the teachers also indicated that nurses not being present on school grounds were a barrier to asthma management.

Sapien et al. (2004) [18]	Asthma knowledge increased postintervention. Teachers felt better recognizing asthma symptoms and administering medication. A school nurse was not always on school grounds. Teachers were not trained. Nurses were not always on school grounds.
